# Personalized Interventional Surgery of the Lumbar Spine: A Perspective on Minimally Invasive and Neuroendoscopic Decompression for Spinal Stenosis

**DOI:** 10.3390/jpm13050710

**Published:** 2023-04-23

**Authors:** Kai-Uwe Lewandrowski, Anthony Yeung, Morgan P. Lorio, Huilin Yang, Jorge Felipe Ramírez León, José Antonio Soriano Sánchez, Rossano Kepler Alvim Fiorelli, Kang Taek Lim, Jaime Moyano, Álvaro Dowling, Juan Marcelo Sea Aramayo, Jeong-Yoon Park, Hyeun-Sung Kim, Jiancheng Zeng, Bin Meng, Fernando Alvarado Gómez, Carolina Ramirez, Paulo Sérgio Teixeira De Carvalho, Manuel Rodriguez Garcia, Alfonso Garcia, Eulalio Elizalde Martínez, Iliana Margarita Gómez Silva, José Edgardo Valerio Pascua, Luis Miguel Duchén Rodríguez, Robert Meves, Cristiano M. Menezes, Luis Eduardo Carelli, Alexandre Fogaça Cristante, Rodrigo Amaral, Geraldo de Sa Carneiro, Helton Defino, Vicky Yamamoto, Babak Kateb, on behalf of Teams/Organizations/Institutions

**Affiliations:** 1 Center for Advanced Spine Care of Southern Arizona, Tucson, AZ 85712, USA; 2 Department of Orthopaedics, Fundación Universitaria Sanitas, Bogotá 111321, Colombia; 3 Department of Orthopedics at Hospital Universitário Gaffree Guinle Universidade Federal do Estado do Rio de Janeiro, R. Mariz e Barros, 775-Maracanã, Rio de Janeiro 20270-004, Brazil; 4 Brain Technology and Innovation Park, Pacific Palisades, CA 90272, USA; 5 Desert Institute for Spine Care, 1635 E Myrtle Ave Suite 400, Phoenix, AZ 85020, USA; 6 Department of Neurosurgery, University of New Mexico School of Medicine, 915 Camino de Salud NE Albuquerque, Albuquerque, NM 87106, USA; 7 Advanced Orthopedics, 499 East Central Parkway, Altamonte Springs, FL 32701, USA; 8 Department of Orthopedic Surgery, The First Affiliated Hospital of Soochow University, No. 899 Pinghai Road, Suzhou 215031, China; 9 Minimally Invasive Spine Center Bogotá D.C. Colombia, Reina Sofía Clinic Bogotá D.C. Colombia, Bogotá 110141, Colombia; 10 Spine Clinic, The American-Bitish Cowdray Medical Center I.A.P, Campus Santa Fe, Mexico City 05370, Mexico; 11 Department of General and Specialized Surgery, Gaffrée e Guinle University Hospital, Federal University of the State of Rio de Janeiro (UNIRIO), Rio de Janeiro 20000-000, Brazil; 12 Good Doctor Teun Teun Spine Hospital, Seoul 775 , Republic of Korea; 13 Torres Médicas Hospital Metropolitano, San Gabriel y Nicolás Arteta Torre Médica 3, Piso 5, Quito 170521, Ecuador; 14 DWS Spine Clinic Center, CENTRO EL ALBA-Cam. El Alba 9500, Of. A402, Región Metropolitana, Las Condes 9550000, Chile; 15 Department of Orthopaedic Surgery, Faculdade de Medicina de Ribeirão Preto (FMRP) da Universidade de São Paulo (USP), Ribeirão Preto 14040-900, Brazil; 16 Hospital Obrero N°1, C. Lucas Jaimes 76, La Paz 0201-0220, Bolivia; 17 Department of Neurosurgery, Spine and Spinal Cord Institute, Gangnam Severance Hospital, Yonsei University College of Medicine, Seoul 731, Republic of Korea; 18 Department of Neurosurgery, Nanoori Hospital Gangnam Hospital, Seoul 731, Republic of Korea; 19 Department of Orthopaedic Surgery, West China Hospital Sichuan University, Chengdu 610041, China; 20 Department of Orthopaedic Surgery, The First Affiliated Hospital of Soochow University, Suzhou 215005, China; 21 Santa Fe Foundation and Instituto Roosevelt, Bogotá 110311, Colombia; 22 Centro de Cirugía Mínima Invasiva—CECIMIN, Avenida Carrera 45 # 104–76, Bogotá 0819, Colombia; 23 Department of Neurosurgery, Pain and Spine Minimally Invasive Surgery Service at Gaffree Guinle University Hospital, Rio de Janeiro 20270-004, Brazil; 24 Department of Orthopaedic Surgery, Espalda Saludable, Hospital Angeles Tijuana, Tijuana 22010, Mexico; 25 Department of Spine Surgery, Hospital de Ortopedia, UMAE “Dr. Victorio de la Fuente Narvaez”, Ciudad de México 07760, Mexico; 26 Department of Spine Surgery, Hospital Ángeles Universidad, Av Universidad 1080, Col Xoco, Del Benito Juárez, Ciudad de México 03339, Mexico; 27 Department of Neurosurgery, Palmetto Steward General Hospital, South Miami, FL 33143, USA; 28 Center for Neurological Diseases, Bolivian Spine Association, Spine Chapter of Latin American Federation of Neurosurgery Societies, Public University of El Alto, La Paz 0201-0220, Bolivia; 29 Santa Casa Spine Center, São Paulo 09015-000, Brazil; 30 Universidade Federal de Minas Gerais (UFMG), Belo Horizonte 31270-901, Brazil; 31 Instituto Nacional Traumato-Ortopedia-INTO, Rio de Janeiro 20940-070, Brazil; 32 Department of Orthopaedic Surgery, University of São Paulo (USP), São Paulo 05508-060, Brazil; 33 Instituto de Patologia da Coluna (IPC), Faculdade de Medicina de Ribeirão Preto (FMRP) da Universidade de São Paulo (USP), São Paulo 14040-900, Brazil; 34 Department of Orthopaedic Surgery, Hospital da Restauração, Recife 52171-011, Brazil; 35 Hospital das Clínicas of Ribeirao Preto Medical School, Sao Paulo University, Ribeirão Preto 14040-900, Brazil; 36 The USC Caruso Department of Otolaryngology-Head and Neck Surgery, USC Keck School of Medicine, Los Angeles, CA 90033, USA; 37 USC-Norris Comprehensive Cancer Center, Los Angeles, CA 90033, USA; 38 World Brain Mapping Foundation (WBMF), Pacific Palisades, CA 90272, USA; 39 Society for Brain Mapping and Therapeutics (SBMT), Pacific Palisades, CA 90272, USA; 40 National Center for Nano Bio Electronic (NCNBE), Los Angeles, CA 90272, USA

**Keywords:** lumbar spinal stenosis, herniated disc, pain generators, staged-management, transforaminal endoscopic surgery, translaminar minimally invasive surgery, guideline

## Abstract

Pain generator-based lumbar spinal decompression surgery is the backbone of modern spine care. In contrast to traditional image-based medical necessity criteria for spinal surgery, assessing the severity of neural element encroachment, instability, and deformity, staged management of common painful degenerative lumbar spine conditions is likely to be more durable and cost-effective. Targeting validated pain generators can be accomplished with simplified decompression procedures associated with lower perioperative complications and long-term revision rates. In this perspective article, the authors summarize the current concepts of successful management of spinal stenosis patients with modern transforaminal endoscopic and translaminar minimally invasive spinal surgery techniques. They represent the consensus statements of 14 international surgeon societies, who have worked in collaborative teams in an open peer-review model based on a systematic review of the existing literature and grading the strength of its clinical evidence. The authors found that personalized clinical care protocols for lumbar spinal stenosis rooted in validated pain generators can successfully treat most patients with sciatica-type back and leg pain including those who fail to meet traditional image-based medical necessity criteria for surgery since nearly half of the surgically treated pain generators are not shown on the preoperative MRI scan. Common pain generators in the lumbar spine include (a) an inflamed disc, (b) an inflamed nerve, (c) a hypervascular scar, (d) a hypertrophied superior articular process (SAP) and ligamentum flavum, (e) a tender capsule, (f) an impacting facet margin, (g) a superior foraminal facet osteophyte and cyst, (h) a superior foraminal ligament impingement, (i) a hidden shoulder osteophyte. The position of the key opinion authors of the perspective article is that further clinical research will continue to validate pain generator-based treatment protocols for lumbar spinal stenosis. The endoscopic technology platform enables spine surgeons to directly visualize pain generators, forming the basis for more simplified targeted surgical pain management therapies. Limitations of this care model are dictated by appropriate patient selection and mastering the learning curve of modern MIS procedures. Decompensated deformity and instability will likely continue to be treated with open corrective surgery. Vertically integrated outpatient spine care programs are the most suitable setting for executing such pain generator-focused programs.

## 1. Introduction and Method:

Lumbar spinal stenosis (LSS) is defined as a narrowing of the lumbar spinal canal caused by age-related degeneration of the spinal motion segment. It causes encroachment of neural elements and may contribute to back and leg pain. It is a leading cause of disability from lack of mobility due to neurogenic claudication worldwide [1]. Spinal stenosis decompression has been shown to reduce claudication-related disability and improve quality of life [2]. In older people, spinal stenosis decompression has become the most common surgical indication. Approximately 600,000 lumbar decompression surgeries are performed annually in the United States alone [3]. Complex spinal fusion surgeries have also substantially increased. From 2002 to 2007, there has been a 15-fold increase in the complex fusion rate [4]. The latter study found that the cost associated with these surgeries in Medicare beneficiaries has risen substantially, totaling an estimated USD 1.65 billion in 2007 [5]. During the same period, the lumbar spine surgery operation rates increased from 1.3 to 19.9 per 100,000 Medicare beneficiaries. This trend was also accompanied by a jump in life-threatening complications from 2.3% to 5.6% due to the proportion of patients being treated with complex fusions. As a result, unplanned hospital readmissions within 30 days from the index operation have risen from 0.8% in lumbar decompression versus 13.0% in complex fusion patients, with a higher adjusted mean hospital charge of USD 80,888 for the latter compared with USD 23,724 for the former [4]. One study illustrated the regional variations in lumbar fusion surgery rates and a 500% spending increase from USD 75 million in 1992 to USD 482 million in 2003 [6].

Spondylolisthesis [7] and decompression-induced iatrogenic instability [8] indicate lumbar spinal fusion. Modern minimally invasive and endoscopic decompression surgery (MIS) is increasingly being used as a less burdensome and simplified alternative to more traditional open decompression techniques where reoperations of one in five patients within three years are commonplace [9]. While a formal prospective cohort study is currently underway comparing lumbar targeted MIS decompression to open decompression and fusion [10], existing studies suggest that lumbar endoscopic decompression stenosis in the central and lateral canal is associated with a low long-term fusion rate of 2.7% [11] in one study and 8.9% in another [12]. A large body of literature with corroborating results demonstrating its cost-effectiveness [13,14,15,16] reported favorable clinical outcomes with minimal targeted decompression for lumbar bony and soft tissue stenosis. It has been published within the last five years [17,18]. More surgeons implement these procedures into their surgical practice portfolio [19]. One study even reported that the endoscopic surgery platform is now the preferred MIS performed in the lumbar spine and is more popular than tubular retractor-based microsurgical decompression surgeries [20]. 

### Method

In this review perspective article, the authors take a fresh, consensus look at the available evidence including their own professional expertise and experiences on using minimally invasive and endoscopic lumbar decompression techniques in a more targeted and personalized care model. 14 professional societies reviewed recent articles on the topic and through their related committees and subcommittees express their professional position. This perspective paper is coordinated by the Interamerican Society For Minimally Invasive Spine Surgery–La Sociedad Interamericana de Cirugía de Columna Mínimamente Invasiva (SICCMI) and the Spine Subcommittee of the Society For Brain Mapping & Therapeutics (SBMT), and endorsed by the International Society For Minimal Intervention In Spinal Surgery (ISMISS), the Korean Minimally Invasive Spine Society (KOMISS), the Minimally Invasive Surgery Section of the Chinese Orthopaedic Association (COA-MIS SECTION), The Colombian Spine Society, the Bolivian Spine Association, the Iberolatinoamerican Spine Society–La Sociedad Iberolatinoamericana de Columna (SILACO), the Mexican Association of Spinal Surgeons–Associacion Mexicana de Cirujanos de Columna (AMCICO), the Federation of Latinamerican Neurosurgical Societies–Federación Latino-Americana de Sociedades de Neurocirugía (FLANC), the Latin American Society of Neurosurgeons of USA & Canada (SLANC), the Brazilian Spine Society–Sociedade Brasiliiera de Columna (SBC), the Brazilian Society For Thoracic Surgery—Sociedade Brasileira de Cirurgia Torácica (SBCT), and the International Intradiscal Therapy Society (IITS).

## 2. Disease Burden

**Figure 1:**
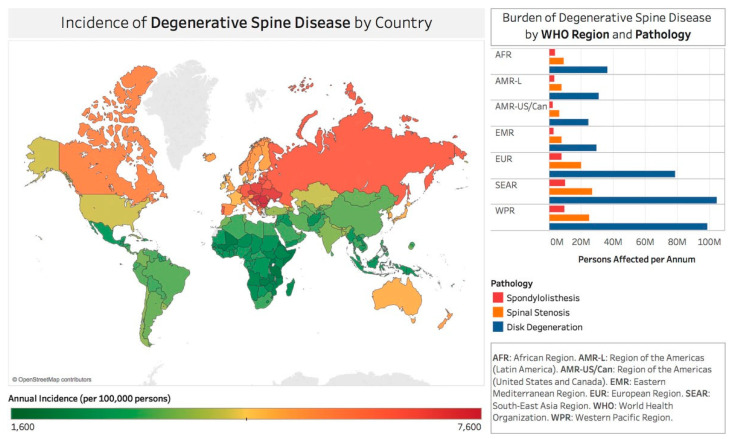
World map depiction of the incidence rates of degenerative spine disease for countries recognized by the World Bank and World Health Organization. Reproduced with permission from Ravindra V et al. [21].

The annual incidence of adult spine disease is estimated at 266 million people globally [21]. The highest per capita yearly incidence is in Europe (5668 per 100,000) and North America (4501 per 100,000) [21]. Based on regional population variations, Southeast Asia and the Western Pacific have the highest volume of patients suffering from painful degenerative spine diseases, with 69 and 65 million people, respectively [21]. The healthcare systems in low- and mid-income countries encounter nearly four times as many total patients as in high-income countries. These estimates are likely hampered by inaccurate reporting terminology. On an annualized basis, 39 million individuals (0.53%) worldwide are diagnosed with spondylolisthesis [21]. Europe has the highest estimated incidence (0.83%), and Africa has the lowest (0.36%). A recent meta-analysis indicated that the incidence of spondylolisthesis and LBP is about 3.5 times higher in low-income countries, suggesting that approximately 400 million people are diagnosed with painful disc degeneration worldwide every year [21]. Globally, the incidence rate is 5.5%. In Europe, it is estimated at 8.6%, whereas in Africa, it is the lowest at 3.7% [21]. Low- and middle-income countries have nearly 3.5 times the incidence of disc degeneration and LBP than high-income countries. Spinal stenosis and low back pain are diagnosed yearly in 102 million individuals worldwide (1.4%). Again, the highest estimated incidence is found in Europe (2.2%) and the lowest in Africa (0.94%), with a similar ratio of 3.5 times greater incidence in low- and middle- versus high-income countries [21]. These geographic disparities likely represent differences in socioeconomic status and access to medical care (Figure 1).

In the 2010 Global Burden of Disease (GBD) Study [22,23], LBP was associated with the was ranked highest rate of years lost to disability among the 291 studied conditions. Eighty-three million disability-adjusted life years (DALYs) were lost to LBP in 2010. A 2019 systematic analysis of the global burden of 369 diseases and injuries in 204 countries and territories analyzing changes in disease burden from 1990–2019 showed that low back pain-related symptoms were the fourth most common cause of disease in the top ten for the 10–49-year age group [24].

## 3. Medical Necessity Criteria

Most contemporary medical necessity criteria for spinal decompression are based on analyzing advanced imaging studies such as MRI or CT. Using these image-based criteria reserves decompression surgery only for patients with progressive disease [25,26,27,28,29,30,31,32,33,34,35,36,37,38,39,40]. By their very definitions, these criteria almost always invite aggressive open surgeries at the end stage of the disease. The net effect is often that patients have to wait until they meet these advanced disease criteria, discounting that most spine care is driven by treating primary pain generators that cause most of the patient’s functional disability. This functional impairment may vary considerably in older patients who often display multilevel disease on MRI or CT but suffer from single-level or unilateral radiculopathy or claudication symptoms. Image-based descriptors of stenosis, deformity, and instability are the key elements indicating traditional open spine surgery.

Whoever does not fit these medical necessity criteria is often committed to repetitive cycles of spinal injections, physical therapy, and non-steroidal anti-inflammatory treatments. Those who are so disabled that they cannot even participate in these programs in a meaningful way or have poorly controlled co-morbidities which preclude medical management often cannot get much help. An exemplary summary of traditional Review Clinical Guidelines For Spinal Stenosis affecting coverage decisions is shown in Table 1 substantiating the reliance on image criteria. In the case of discordant reading between surgeon and radiologist that is unresolvable following review, the reference exemplary protocol calls for another independent radiologist review of the studies, rather than for a clinical context care model to identify the responsible pain generator.

In this perspective paper, SICCMI, in collaboration with 12 other international surgeons’ societies, is confronting the increased scrutiny on the appropriateness of spine care spending by payers, government, and patients in an attempt to deliver on simplified, innovative, less costly, more effective, and more durable treatments for common degenerative and painful spine conditions associated with lower complication and revision rates. Resolution to these problems is needed to meet the increasing demand by the aging baby-boomer population for such simplified treatments.

## 4. Timing of Intervention

The treatment of the painful degenerative disease process of the spinal motion segment with traditional open spine surgery is dictated by the application of MRI and CT image criteria of spinal stenosis, instability, and deformity. The clinical decision-making for surgery focuses on treating the end-stage of the disease, leaving many patients without timely treatment or no treatment at all. With the backdrop of recent clinical studies describing the limited utility of the lumbar MRI scan and its reporting suffering from relatively low sensitivity and specificity to diagnose the painful spine condition [42], key opinion leaders of the sponsoring and endorsing societies took the position that it is time to rethink traditional clinical management protocols for sciatica-type back and leg pain. The continued application of outdated image-based medical necessity criteria is not suitable for the cost-effective spine care needed for the future. This perspective paper highlights the concepts of pain generators in the lumbar spine that are frequently trivialized as seemingly minor conditions. Examples include annular tears, inflamed or tethered nerve roots, small extraforaminal disc herniations under the dorsal root ganglion, which can chronically in-flame it and cause leg pain, or impaction syndromes of the facet joint complex, which can form painful, highly inflammatory extradural synovial cysts. These inflammatory and painful conditions in the aging lumbar spinal motion segment can cause severe symptoms that may appear out of proportion and unsupported by the definitions of the routine lumbar MRI scan and its reporting [42]. Treating the structural correlates of lumbar spine disease early to reduce the long-term disability associated with the disease by healing painful conditions and altering their natural history if left untreated is the position the authors of this perspective article are taking on behalf of their respective societies.

## 5. Standards

Traditional open spine surgery in the lumbar spine includes laminectomy. A translaminar decompression is performed by removing the posterior spinal elements, including the spinous process, the lamina, and part of the bilateral facet joints. The benefit of lumbar decompression for symptomatic herniated disc and spinal stenosis has been demonstrated in several prospective randomized clinical trials, including the original study by Weber [43], the Main Lumbar Spine Study [44,45,46], and the Spine Outcome Research Trial (SPORT) [7,47,48,49,50,51,52]. Multiple investigators corroborated these observations and illustrated the cost-effectiveness of surgical decompression over conservative care consisting of active therapy-based, interventional, and medical management programs [53,54,55,56].

## 6. Minimally Invasive Spine Surgery

Minimally invasive surgery (MIS) for the treatment of common degenerative conditions of the spine is increasingly practiced. It encompasses a portfolio of surgical techniques that are aimed to alleviate patients’ pain via reduced surgical access resulting in less blood loss and reduced peri- and postoperative problems including pain, nausea, and vomiting. The introduction of tubular retractors and microsurgical dissection techniques simplified the very nature of spine surgery by transforming it through the implementation of less burdensome and more simplified protocols. Over the last 40 years, open spine surgery has established a track record that is interpreted by most patients, employers, and payers as aggressive and too costly due to high out-of-work complications and reoperation rates.

## 7. Cost Effectiveness of MIS

Quality of life and cost-effectiveness of minimally invasive surgery (MIS) for spinal stenosis in patients with degenerative lumbar spondylolisthesis (DLS) has been calculated relative to failed medical management and compared to the cost-effectiveness of hip and knee arthroplasty for matched cohorts of patients with osteoarthritis [54]. Incremental cost–utility ratios (ICUs) were calculated from utilization cost and quality-adjusted life-years (QALYs) data. Surgical treatment resulted in faster recovery from sciatica, making early surgery more cost-effective than prolonged conservative care. With a calculated QALY of EUR 40,000, withholding lumbar decompression surgery was not found to be cost-effective even when adjusting for missed work hours [54].

## 8. Pain Generators

Select pain generators in the lumbar spine are not diagnosed on routine lumbar MRI scan [42]. A recent study showed that nearly half of the surgically treated pain generators were not shown by the preoperative MRI scan (Table 2) [57].

**Figure 2:**
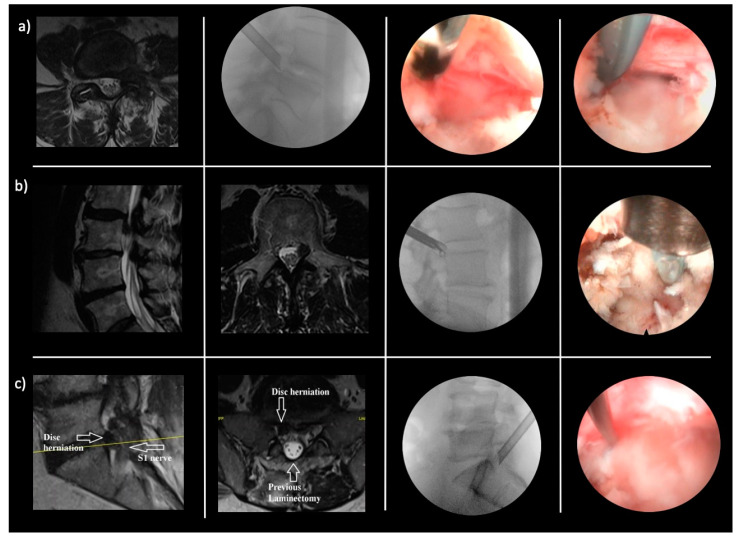
Common examples of pain generators missed by the routine lumbar MRI scan: (**a**) Inflamed nerve. Left to right: T2 axial MR image demonstrating the severe left L4–5 foraminal stenosis and foraminal disc herniation. Lateral fluoroscopic image of the beveled tubular retractor in the left L4–5 foramen. Endoscopic camera view of the inflamed left L5 traversing nerve root not demonstrated on MRI scan. Endoscopic camera view of the radiofrequency probe elevating the traversing left L5 never root and revealing the compressing disc fragment below. (**b**) Nerve tethering. Left to right: T2 sagittal MR image demonstrating the caudally extruded disc fragment behind the body of L3. T2 MR axial image of the left L2–3 caudally extruded disc fragment medial to the left L3 pedicle. Lateral fluoroscopic image of the bendable endoscopic grasper removing the caudally extruded disc fragment. Endoscopic camera view of the extruded disc fragment visible after removing the cranial portion of the L3 pedicle. A radio frequency probe is demonstrated above the L3 pedicle. The tethering of the nerve root by scar tissue was missed by the MRI scan. (**c**) Hypervascular scar. Left to right: T2 sagittal MR image demonstrating the L5-S1 disc herniation compressing the S1 nerve. T2 MR axial image demonstrating the L5-S1 disc herniation and the significant laminectomy defect. Lateral fluoroscopic images demonstrate the position of the beveled 7 mm tubular retractor in the right L5-S1 foramen. Endoscopic camera view of the endoscopic ball probe used to dissect the S1 nerve off the disc herniation. The MRI scan failed to show the severe hypervascular scar that had to be dissected and coagulated to release the S1 nerve and remove the herniated disc.

Common pain generators in the lumbar spine include (a) an inflamed disc, (b) an inflamed nerve, (c) a hypervascular scar, (d) a hypertrophied superior articular process (SAP) and ligamentum flavum, (e) a tender capsule, (f) an impacting facet margin, (g) a superior foraminal facet osteophyte, (h) a superior foraminal ligament impingement, (i) a hidden shoulder osteophyte, and many others examples, as shown in Figure 2 [58]. The position of the key opinion authors of the perspective article is that further clinical research will continue to validate pain generator-based treatment protocols for lumbar spinal stenosis. The endoscopic technology platform enables spine surgeons to directly visualize pain generators, forming the basis for more simplified targeted surgical pain management therapies.

**Figure 3:**
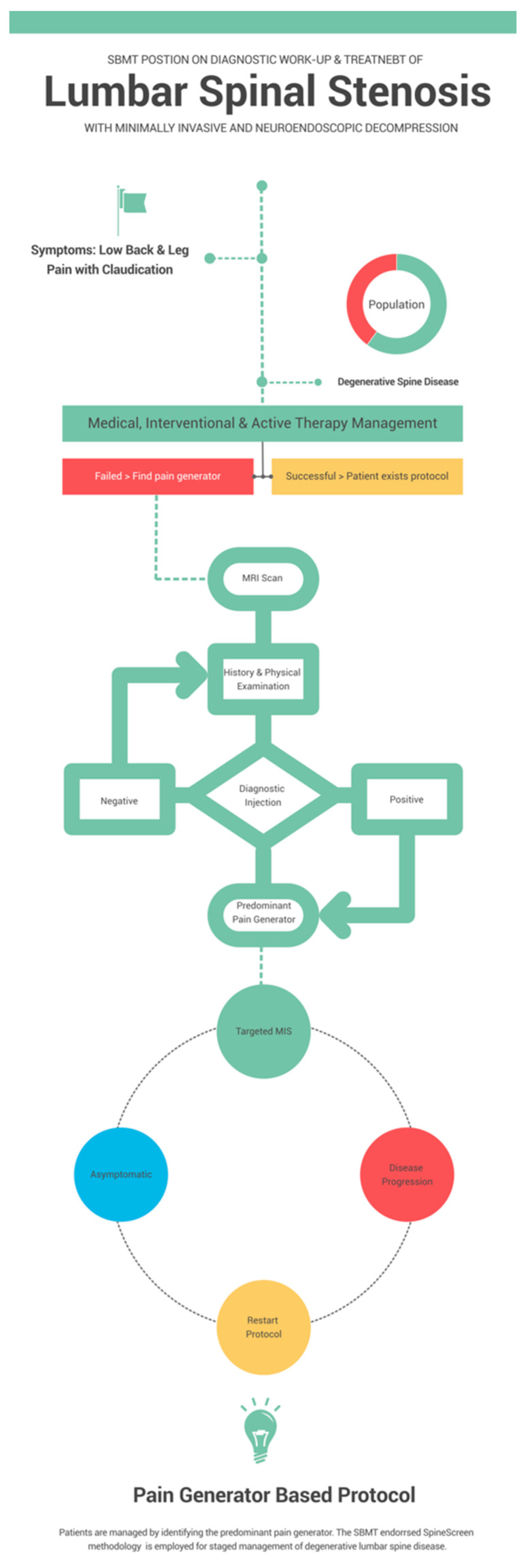
Schematic of endorsing Spine Societies’ work-up and treatment protocol of symptomatic lumbar spinal stenosis due to degenerative spine disease without instability or deformity employing the SpineScreen methodology [57].

One example is highlighted by the INTRACEPT prospective, open-label, 1:1 randomized controlled trial. This trial tests the efficacy of basivertebral nerve (BVN) ablation technology compared to a standard care control treatment of vertebrogenic chronic low back pain [59]. Conceptually, this technology is an example of simplified spine care targeting a validated pain generator. However, the procedure is non-visualized. Instead, the authors of this perspective article call for targeted treatments of painful conditions of the lumbar spine based on the direct visualization of validated pain generators. The authors expect these concepts to ultimately transition into a new hybrid subspecialty—surgical pain management—where interventional pain management doctors and spine surgeons compete for patients [60,61,62,63]. Similar trends have been observed with the creation of interventional radiology or cardiology, where newer protocols have successfully replaced open heart surgery. The SpineScreen protocol [57] is summarized in the schematic shown in Figure 3.

## 9. Direct Visualization

The authors take the position that direct visualization and treatment of pain generators is the foundation for surgical pain management of the lumbar spine. Spinal endoscopy enables the surgeon to diagnose and treat the painful condition during the same operation [64,65,66,67,68]. The ability to directly and videoendoscopically visualize painful pathology has gone chiefly unnoticed by traditionally trained spine surgeons. Limiting the surgical treatment to one major pain generator in the relevant clinical context at the time of treatment is the cornerstone of modern endoscopic spine care. The direct visualization of the epidural space and intervertebral disc opens the door to analysis of the myriad of unrecognized pain generators that frequently escape diagnosis via the routine lumbar MRI scan and its limited reporting of neural element compression by noting the presence or absence of stenosis.

Objective measurements of the foraminal or lateral canal dimensions are rarely given. Surgical stenosis classifications use measurable parameters such as height and width of the posterior disc, lateral recess, or neuroforamen to stratify patients for the most appropriate MIS approach and technique [42]. The lack of accurate MRI prognosticators of favorable clinical outcomes with lumbar decompression surgery illustrates the fundamental limitations of clinical treatment protocols primarily based on image-based criteria rather than accurately triaging patients for pain generators to treat them successfully. Modern spinal endoscopy technology can produce high-resolution videos and images of painful lumbar pathology. When combined with intraoperative examination of the visualized pain generators, correlating them with the patient’s symptoms becomes feasible [68]. The patient’s response to provocative or analgesic testing of structural correlates suspected of causing the patient’s pain allows the spine surgeon to identify the primary pain generator correctly and to effectively treat it with a targeted procedure.

## 10. Staged Management

Treating pain generators involves identifying those structural problems in the lumbar spine that cause the majority of the patient’s symptoms as implicated in the symptoms by history and physical examination or by advanced imaging studies such as MRI or CT scan. Most patients have unilateral or mono-segmental radiculopathy symptoms. When confirmed with diagnostic selective nerve root blocks, these clinical observations highlight that many structural changes in a degenerative spine are multilevel but may not be painful, even if the MRI or CT scan suggests a similar degree of stenosis as within the symptomatic spinal motion segment. For example, exiting and traversing nerve root pain syndromes may or may not exist within the same lumbar motion segment simultaneously. However, when they do, the surgeon can easily be confused with patients whose MRI suggests multilevel disease. Identifying the correct source level of axial facet joint pain in multilevel degeneration may even be more difficult without a radicular component. The staged management concept is the central element of the clinical spine care model. It implies treating validated symptomatic pain generators and ignoring all degenerative changes or injuries that do not hurt.

While it is easier to rely on MRI-based criteria of compression, instability, and deformity when deducting a plan of surgical care—certainly within the mainstream of traditionally trained surgeons, and perhaps a coincidence between the radiologist’s confirmation of compressive pathology and the surgical plan of care invites less scrutiny during the health insurance preauthorization process for surgery—identifying the predominant pain generator can be a daunting task. Recommending a targeted surgical treatment plan to a patient in pain is as much of an art as it is a science and relies heavily on judgment and clinical experience. Less aggressive treatment recommendations are often sufficient to substantially reduce pain, all while ignoring traditional decompression and lumbar fusion criteria.

What to treat and, more importantly, what to ignore requires attention to detail and utilization of preoperative diagnostic tools of high positive predictive value. For the endoscopic spine surgeon, the plan of care is derived from identifying those pain generators that impair the patient the most. Minimizing the risk-taking and maximizing the benefit, all while managing patients’ expectations about the desired outcome, are the key to achieving high patient satisfaction [69]. The staged management approach has been developed and employed by this perspective paper’s principal authors to emphasize a care plan rooted in identifying pain generators limiting the patients’ functioning when spine care is delivered. The peer-reviewed and published literature by KOL authors proves that this approach to endoscopic spine care results in favorable long-term clinical outcomes up to five [11,12] and ten years post-operatively [57]. Reoperations at the index location are uncommon, and additional decompression and ablation surgeries are typically performed within the same level on the other side or at the adjacent level [70,71,72,73]. Traditional open decompression fusion surgeries are rarely needed [68,74]. Occasionally, undertreatment with unchanged or reduced persistent symptoms due to failure to cure may arise when employing the staged management approach to the surgical treatment of lumbar spinal stenosis [75]. However, these patients can often easily be managed by reanalyzing their pain generators with the protocol depicted in figure three and performing the additional targeted endoscopic surgeries if necessary. This management style makes it possible to overcome functional limitations when they become relevant to the patient. Shared decision-making with the patient is the authors’ preferred methodology of engaging patients and having them partake in their spine care. Treating the consequences of surgical overtreatment with persistent or new pain arising from well-established adjacent segment disease problems or failed surgeries is much more challenging [76,77,78]. Sometimes the latter is impossible. Patient satisfaction and motivation to continue with their surgeon typically remain high even with incomplete symptom resolution when shared decision-making occurs preoperatively. In the authors’ experience, inappropriate point-of-care transitions and overutilization are rare.

## 11. Surgical Pain Management

A new subspecialty is emerging: “Surgical Pain Management.” The term implies a blend of diagnostic and patient management strategies employed by interventional pain physicians comprised of physiatrists, anesthesiologists, and spine surgeons consisting of orthopedic and neurosurgeons. This new emerging subspeciality is a grassroots development driven by enthusiast physicians who invested their careers into a more personalized approach to spine care. Thus, surgical pain management integrates needle-based non-visualized interventions into MIS and endoscopic surgical procedures by tailoring the treatment based on the individual patient’s symptoms and the functional context when the spine care is delivered. Examples of this development include the integration of radiofrequency and laser into endoscopic surgeries. The continued use of rule-based medical necessity criteria for lumbar spine care seems increasingly inappropriate. It invites the delivery of costly, ineffective therapies and treatments which ultimately do not lower the societal burden of spine care. Ignoring individual pain generators stemming from the underlying disease causing cumulative disability does not address the root cause and precludes the patient from definitive care or leads to patient entrapment in repetitive yet ineffective treatment cycles. In the opinion of the KOL authors of this lead perspective article in the JPM special issue “The Path To Personalized Pain Management,” the staged approach to surgical pain management is poised to lower disability and the direct and indirect costs with all of its hidden unintended consequences of repetitive treatments. Its implementation has the potential for diminishing the burden of failed medical pain management and opioid addiction, delayed returned to work, and disrupted social reintegration. While the authors do not suggest abandoning traditional open spine surgery, we propose integrating staged surgical pain management of directly visualized pain generators early in the disease process into existing spine care programs.

## 12. Discussion

The authors of this perspective paper arrived at the consensus statement on the indications for surgical treatment for symptomatic lumbar spinal stenosis based on validated pain generators rather than traditional image-based medical necessity criteria. This approach to treating patients with claudication and sciatica-type low back and leg symptoms represents the authors’ views and their respective surgeon societies. The most published individuals on this team of authors are accomplished and passionate endoscopic spinal surgeons. In experienced hands, approximately 80% of patients with painful degenerative spine conditions may be treated successfully with a targeted outpatient MIS or endoscopic decompression procedure [66,79,80,81,82,83]. When the staged management protocol is employed as described in this perspective article, open surgery may only be required in a minority of patients with severe multilevel lateral stenosis, painful facet disease, high-grade instability, and deformity. The strength of MIS and endoscopic surgery techniques may also be its most significant limitation: the focused surgical care through a small incision. An adequate decompression or reconstruction may not be possible in patients with advanced disease. Those patients are best treated with a traditional index decompression fusion surgery where large spine sections can be appropriately decompressed and reconstructed with attention to instability or sagittal and coronal alignment correction. The discussion on the most appropriate application of modern MIS and endoscopic spinal surgery techniques will likely continue and be driven by technological advances.

## 13. Conclusions

Judiciously and skillfully executed modern targeted MIS surgeries can provide more cost-effective and less burdensome spine care with shorter treatment cycles as the underlying structural correlate for the patient’s pain is treated causally. Lateral recess and foraminal stenosis are the most common clinically relevant indications for primary surgery and revision surgery after decompression in the lumbar spine. The personalized clinical protocols for treating lumbar spinal stenosis based on validated pain generators are a break with traditional population management protocols that employ image-based medical necessity criteria for intervention since nearly half of the surgically treated pain generators are not shown on the preoperative MRI scan. Common pain generators in the lumbar spine include (a) an inflamed disc, (b) an inflamed nerve, (c) a hypervascular scar, (d) a hypertrophied superior articular process (SAP) and ligamentum flavum, (e) a tender capsule, (f) an impacting facet margin, (g) a superior foraminal facet osteophyte, (h) a superior foraminal ligament impingement, (i) a hidden shoulder osteophyte. It is the authors position, that further clinical research will continue to validate pain generator-based treatment protocols for lumbar spinal stenosis. The endoscopic technology platform enables spine surgeons to directly visualize pain generators, forming the basis for more simplified targeted surgical pain management therapies. Clinical judgment of appropriate patient selection and mastering the learning curve of modern MIS procedures will likely lead to a broadening of the accepted indications for such a pain generator-based approach to patient care. Limitations of this care model are dictated by decompensated deformity and instability, for which open corrective surgery will likely continue to be the treatment of choice. Vertically integrated outpatient spine care programs are probably the most suitable setting for executing such a pain generator focused program since physicians who have complete custody of their patients can minimize protocol breaches by other providers who are not invested in the personalized care model proposed by the authors.

## Figures and Tables

**Table 1:** Exemplary Review Criteria for Lumbar Spinal Stenosis Surgery Affecting Coverage Decisions cited from Washington State Department of Labor and Industries Surgical Guideline for Lumbar Spine–September 2021 [41].

A Request May Be Appropriate for	If the Patient Has	AND the Diagnosis Is Supported by These Clinical Findings:		And This Has Been Done	
Surgical Procedure	Condition or Diagnosis	Subjective	Objective	Imaging	Non-Operative Care
Lumbar Decompression including: Lumbar laminectomy, laminotomy, discectomy, microdiscectomy, foraminotomy, or far lateral decompression	Nerve Root Entrapment due to central/paracentral/foraminal/extra-foraminal herniated nucleus pulposus.	Sensory symptoms in dermatomal distribution including: Radiating pain, burning, numbness, tingling, or paresthesia.	Objective findings must include two or more of the following: Dermatomal sensory deficit on exam. Motor deficit (e.g., foot drop or quadriceps weakness). Positive dural tension signs (e.g., straight leg test, contralateral straight leg test/crossover sign). Asymmetric reflex changes. Positive EMG demonstrates acute denervation (fibrillation and sharp waves) corresponding with the level of intended surgery. -Reproduction of back pain alone is not a positive finding.	CT-Myelogram or MRI (within 6 months of requested surgery) must corroborate subjective and objective findings with substantial disc herniation, resulting in one or more of the following on the nerve root: Effacement Abutment Displacement Compression Stenosis Mild to moderate disc protrusion not associated with the above terms is not considered a positive objective imaging sign. In the case of discordant reading between surgeon and radiologist that is unresolvable following review, another independent radiologist review is required.	At least six weeks of non-operative care from the date of injury, unless substantial or progressive motor weakness is documented. Care may include: Active rehabilitation Manual medicine Pharmacologic therapy Epidural steroid injection
Lumbar Decompression including: Lumbar laminectomy, laminotomy, or discectomy	Central spinal stenosis, moderate or severe	Neurogenic claudication, defined as: Radiating leg pain that is exacerbated while standing up and walking. Immediate relief of neurogenic symptoms when seated. Improvement of symptoms when bending forward.	Bilateral lower extremity pain or weakness with standing and walking. -If unilateral pain is present, hip or vascular pathology should be ruled out by exam.	MRI or CT-Myelogram (within 6 months of requested surgery) confirms subjective and objective findings of moderate or severe central spinal stenosis. In the case of discordant reading between surgeon and radiologist that is unresolvable following review, another independent radiologist review is required.	At least six weeks of non-operative care from the date of injury, unless substantial or progressive motor weakness is documented. Care may include: Active rehabilitation Manual medicine Pharmacologic therapy Epidural steroid injection

Source: Treatment Guideline for Lumbar Spine Surgery. Washington State Department of Labor and Industries Surgical Guideline for Lumbar Spine–September 2021. Website: https://www.lni.wa.gov/patient-care/advisory-committees/_docs/LumbarSpineSurgeryGuidelineSeptember2021FinalUpdate.pdf (accessed on 7 July 2021).

**Table 2:** Crosstabulation primary pain generator visualized during minimally invasive endoscopic operation and their reporting on routine lumbar MRI.

Endoscopically Visualized Pain Generator	MRI Negative	MRI Positive	Total:
Hypertrophied Ligamentum Flavum	7	35	42
	7.3%	31.8%	20.4%
Contained Herniated Disc	6	25	31
	6.3%	22.7%	15.0%
Hypertrophied Superior Articular Process	3	24	27
	3.1%	21.8%	13.1%
Inflamed Disc With Toxic Annular Tear	25	0	25
	26.0%	0.0%	12.1%
Extruded Herniated Disc	5	19	24
	5.2%	17.3%	11.7%
Delaminated and Fissured Disc Tissue	17	0	17
	17.7%	0.0%	8.3%
Intra-Annular Granulation Tissue	9	0	9
	9.4%	0.0%	4.4%
Facet Cyst	1	7	8
	1.0%	6.4%	3.9%
Hidden Shoulder Osteophyte	7	0	7
	7.3%	0.0%	3.4%
Inflamed Nerve	6	0	6
	6.3%	0.0%	2.9%
Tethered and Furcal Nerve Roots	6	0	6
	6.3%	0.0%	2.9%
Contracted Foraminal Ligaments	4	0	4
	4.2%	0.0%	1.9%
Total ELD Patients	96	110	206
	100.0%	100.0%	100.0%

MRI Negative: The radiologist did not describe the endoscopically visualized primary pain generator in the MRI report. MRI Positive: The radiologist did describe the endoscopically visualized primary pain generator in the MRI report. Reproduced with permission from Lewandrowski K-U, Abraham I, Ramírez León JF, et al. A Proposed Personalized Spine Care Protocol (SpineScreen) to Treat Visualized Pain Generators: An Illustrative Study Comparing Clinical Outcomes and Postoperative Reoperations between Targeted Endoscopic Lumbar Decompression Surgery, Minimally Invasive TLIF and Open Laminectomy. Journal of Personalized Medicine 2022;12:1065.

## Data Availability

The data presented in this study are available on request from the corresponding author.

## Supplementary Materials

The following supporting information can be downloaded at: https://www.mdpi.com/article/10.3390/jpm13050710/s1, Supplementary Document S1: Members of organizations.
