# Supplementary material for: Personalized Interventional Surgery of the Lumbar Spine: A Perspective on Minimally Invasive and Neuroendoscopic Decompression for Spinal Stenosis
Source: J Pers Med. 2023 Apr 23;13(5):710. doi: 10.3390/jpm13050710 (PMC10219192; doi:10.3390/jpm13050710)
Supplement: Supplementary file 1 [file jpm-13-00710-s001.zip › jpm-2225455-supplementary.pdf]

Supplementary Document S1.

Members of Interamerican Society For Minimally Invasive Spine Surgery- Sociedad Interamericana de Cirugía de Columna Mínimamente Invasiva (SICCMI)
Kai-Uwe Lewandrowski
Morgan P. Lorio
Álvaro Dowling
Paulo Sérgio Teixeira De Carvalho
Luis Miguel Duchén Rodríguez

Members of the International Society For Minimal Intervention In Spinal Surgery (ISMISS)
Kai-Uwe Lewandrowski

Members of the International Intradisccal Therapy Society (IITS)
Kai-Uwe Lewandrowski
Anthony Yeung

Members of the Society for Brain Mapping and Therapeutics (SBMT)
Kai-Uwe Lewandrowski
Vicky Yamamoto
Babak Kateb

Members of the Chinese Orthopaedic Association–Minimally Invasive Surgery Section (COA-MIS Section)
Huilin Yang
Jiancheng Zeng
Bin Meng

Members of the Iberolatinoamerican Spine Society–Sociedad Iberolatinoamericana de Columna (SILACO)
Jorge Felipe Ramírez León
Jaime Moyano
José Antonio Soriano Sánchez
Luis Miguel Duchén Rodríguez

Members of the Federation of Latinamerican Neurosurgical Societies–Federación Latinoamericana de Sociedades de Neurocirugía (FLANC)
José Antonio Soriano Sánchez
Luis Miguel Duchén Rodríguez
José Edgardo Valerio Pascua

Members of Brazilian Society For Thoracic Surgery – Sociedade Brasileira de Cirurgia Torácica (SBCT)
Rossano Kepler Alvim Fiorelli

Members of the Korean Minimally Invasive Spine Society (KOMISS)
Kang Taek Lim
Jeong-Yoon Park
Hyeun-Sung Kim

Members of the Bolivian Spine Association
Juan Marcelo Sea Aramayo

Luis Miguel Duchén Rodríguez

Members of the Colombian Spine Society–Sociedad Colombiana de Columna (SOCCOL)
Fernando Alvarado Gómez
Carolina Ramirez

Members of Mexican Association Of Spinal Surgeons, Ac-Associacion Mexicana De Cirujanos De Columna (AMCICO)
Alfonso Garcia
Eulalio Elizalde Martínez
Iliana Margarita Gómez Silva
José Antonio Soriano Sánchez

Members of Latin American Society of Neurosurgeons of USA & Canada (SLANC)
José Edgardo Valerio Pascua

Members of the Brazilian Spine Society (SBC)
Robert Meves
Cristiano M. Menezes
Luis Eduardo Carelli
Alexandre Fogaça Cristante
Rodrigo Amaral
Geraldo de Sa Carneiro
Helton Defino
